# Proteomining-Based Elucidation of Natural Product Biosynthetic Pathways in *Streptomyces*

**DOI:** 10.3389/fmicb.2022.913756

**Published:** 2022-07-11

**Authors:** Darwin Linardi, Weiyi She, Qian Zhang, Yi Yu, Pei-Yuan Qian, Henry Lam

**Affiliations:** ^1^Department of Chemical and Biological Engineering, The Hong Kong University of Science and Technology, Kowloon, Hong Kong SAR, China; ^2^Southern Marine Science and Engineering Guangdong Laboratory (Guangzhou), Hong Kong, Hong Kong SAR, China; ^3^SZU-HKUST Joint PhD Program in Marine Environmental Science, Shenzhen University, Shenzhen, China; ^4^Department of Ocean Science and Hong Kong Branch of Southern Marine Science and Engineering Guangdong Laboratory (Guangzhou), The Hong Kong University of Science and Technology, Kowloon, Hong Kong SAR, China; ^5^Hubei Clinical Center and Key Laboratory of Intestinal and Colorectal Disease, Department of Gastroenterology, Zhongnan Hospital of Wuhan University, School of Pharmaceutical Sciences, Wuhan University, Wuhan, China

**Keywords:** *Streptomyces*, proteomics, synthetic biology, natural product, *Actinobacteria*, biosynthetic gene cluster (BGC), antibiotic

## Abstract

The genus *Streptomyces* is known to harbor numerous biosynthetic gene clusters (BGCs) of potential utility in synthetic biology applications. However, it is often difficult to link uncharacterized BGCs with the secondary metabolites they produce. Proteomining refers to the strategy of identifying active BGCs by correlating changes in protein expression with the production of secondary metabolites of interest. In this study, we devised a shotgun proteomics-based workflow to identify active BGCs during fermentation when a variety of compounds are being produced. Mycelia harvested during the non-producing growth phase served as the background. Proteins that were differentially expressed were clustered based on the proximity of the genes in the genome to highlight active BGCs systematically from label-free quantitative proteomics data. Our software tool is easy-to-use and requires only 1 point of comparison where natural product biosynthesis was significantly different. We tested our proteomining clustering method on three *Streptomyces* species producing different compounds. In *Streptomyces coelicolor* A3(2), we detected the BGCs of calcium-dependent antibiotic, actinorhodin, undecylprodigiosin, and coelimycin P1. In *Streptomyces chrestomyceticus* BCC24770, 7 BGCs were identified. Among them, we independently re-discovered the type II PKS for albofungin production previously identified by genome mining and tedious heterologous expression experiments. In *Streptomyces tenebrarius*, 5 BGCs were detected, including the known apramycin and tobramycin BGC as well as a newly discovered caerulomycin A BGC in this species. The production of caerulomycin A was confirmed by LC-MS and the inactivation of the caerulomycin A BGC surprisingly had a significant impact on the secondary metabolite regulation of *S. tenebrarius*. In conclusion, we developed an unbiased, high throughput proteomics-based method to complement genome mining methods for the identification of biosynthetic pathways in *Streptomyces* sp.

## Introduction

The *Streptomyces* sp. are producers of natural products that are often synthesized by genes in proximity in the genome, collectively referred to as biosynthetic gene clusters (BGCs) (Hopwood, 2007; Medema et al., 2015; Genilloud, 2017). Many of these BGCs share common core genes such as polyketide synthases (PKS) and non-ribosomal peptide synthetases (NRPS), which form an assembly line that elongates the metabolite backbone. The combination of these core genes with various functional groups and structure modifying enzymes in a BGC allows the *Streptomyces* sp. to produce a diverse variety of compounds that are difficult to efficiently produce through chemical synthesis (Hopwood, 2007; Rossiter et al., 2017; Genilloud, 2018).

The democratization of genome sequencing has revealed the variety of BGCs in the genomes of *Streptomyces* spp., which contributes to the recent interest in leveraging this genus for synthetic biology applications (Genilloud, 2018). This led to the increased popularity of genome mining, the identification of novel BGCs in newly sequenced genomes via sequence analysis paired with experimental confirmation of the products of the BGCs. One of the most widely used genome mining tools is antiSMASH, which predicts BGCs in the genome and matches the predicted clusters against a database of previously documented BGCs, allowing for rapid identification of putative BGCs in a newly sequenced genome (Blin et al., 2021). Another important genome mining tool is PRISM which uses machine learning to predict the products produced by the identified BGCs (Skinnider et al., 2015). However, putative BGCs predicted by genome mining are not always specific, and many are not actively expressed or “silent.” In addition, since many BGCs are present in a single genome of *Streptomyces* sp., linking predicted BGCs to their respective metabolites can be challenging.

Complementing genome mining approaches with expression-based analyses, such as transcriptomics or proteomics, can help to focus the search for the BGC(s) responsible for the production of compound(s) of interest by correlating secondary metabolite production levels to gene expression levels (Gubbens et al., 2014). For example, transcriptomics has been performed on *Streptomyces flaveolus* (Qu et al., 2011) and on several strains of *Salinispora pacifica* (Amos et al., 2017) to search for BGCs. However, transcripts have short half-lives in bacteria, making it difficult to determine the optimal time points to capture the shift in expression patterns (Moran et al., 2013). On the contrary, proteins are the actual actors in natural product biosynthesis in real-time; thus, one expects that protein levels should be more reliably correlated with secondary metabolite production (Du and van Wezel, 2018; Ludvigsen and Honoré, 2018). The term “proteomining” was coined by Gubbens et al. (2014) to describe the strategy of identifying potential BGCs by correlating changes in the protein expression pattern, measured by proteomics, with the production of secondary metabolites of interest.

The early proteomining methods targeted the phosphopantetheinyl cofactor of PKS and NRPS (Meluzzi et al., 2008; Bumpus et al., 2009). They utilized the mass shift that occurs due to the ionization-induced release of 4′-phosphopantetheine to identify spectra corresponding to PKS/NRPS peptides linked to this cofactor. The sequence of these peptides was then determined *de novo* and reverse translated to develop primers for the targeted PCR study of the gene. To improve the selectivity for peptides of PKS/NRPS, Meier et al. (2009, 2011) developed probes that bind to the conserved active sites or the phosphopantetheinyl cofactor to selectively pull down expressed PKS and NRPS. In another approach, high molecular weight proteins were isolated from streptomycetes to select for PKS and NRPS complexes (Bumpus et al., 2009; Evans et al., 2011; Chen et al., 2012, 2013a,2013b). However, these approaches are “biased,” being able to detect only NRPS and PKS, and do not cover the whole proteome but have the advantage of not requiring the whole genome sequence. To this end, an unbiased method employing shotgun proteomics to compare the proteomes of mycelia grown on different media was proposed (Gubbens et al., 2014). Using this method, the authors linked the expressed BGCs to antibiotic activity and subsequently identified the biosynthetic pathway of a novel juglomycin antibiotic.

In this work, we exploited the actinomycetes’ characteristic of secondary metabolite BGCs expression during nutrient starvation and developed a label-free proteomics-based method for proteomining. The non-producing, nutrient-rich growth phase was used as background to the secondary metabolite producing, nutrient-starved stationary phase. This served as another axis of comparison alongside comparison between mutant strains or different fermentation media. The contrast between the producing/non-producing proteome states, combined with the clustering of differentially expressed proteins based on proximity in the genome, would pinpoint expressed BGCs. To demonstrate the utility of the method, three *Streptomyces* sp. were studied in this work. First, we applied the method to the well-studied *Streptomyces coelicolor* A3(2) as a proof of concept. Then, we sought to identify active BGCs in *Streptomyces chrestomyceticus* BCC24770 and *Streptomyces tenebrarius*.

## Experimental Methods

### Strain Cultivation Conditions

Fermentation of *S. coelicolor* A3(2) and *S. chrestomyceticus* BCC24770 was performed at 28°C and 250 RPM with 30 glass beads per flask. Fermentation of *S. tenebrarius* was performed at 37°C and 250 RPM. Spores of *S. coelicolor* A3(2) and BCC24770 were activated in GYM broth (4 g glucose, 4 g yeast extract, and 10 g malt extract per liter Milli-Q water, DSMZ Medium 65) for 24 to 48 h (OD_600_ = 1) and transferred to fresh GYM broth (1% inoculum) to start the fermentation. Fermentation of BCC24770 in TSBY broth (30 g tryptic soy broth and 5 g yeast extract per liter of Milli-Q water) media was inoculated using GYM broth culture at OD_600_ = 1 (1% inoculum). Similarly, spores of *S. tenebrarius* were activated in TSBY for 24 h and transferred to fresh TSBY broth. Fermentation of *S. tenebrarius* was performed at 37°C and 250 RPM. All fermentation experiment was performed for up to 3 days. The harvested fermentation liquid was separated by centrifugation and both fractions were stored at −20°C until sample processing.

### Secondary Metabolite Characterization

Undecylprodigiosin was extracted and characterized based on the method previously developed (Tenconi et al., 2013). In summary, the mycelia of *S. coelicolor* A3(2) at 96 h were incubated in acidified methanol (pH < 4) for 1 h. The suspension was centrifuged to remove cell debris. The absorbance spectra of the supernatant were measured using Varioskan™ LUX multimode microplate reader. Undecylprodigiosin-hydrochloride (Sigma Aldrich) was used as standard. Mass spectrometry analysis was performed by direct injection to a SCIEX TripleTOF 4600.

Apramycin concentration in the supernatant was determined by a Waters Acquity HPLC coupled with an evaporative light scattering detector (ELSD) (Lv et al., 2016; Zhang et al., 2021). The column used was Waters CORTECS™ UPLC C18 1.6 μm (2.1 × 100 mm). Mobile phases A and B were 20 mM heptafluorobutyric acid in water and acetonitrile, respectively. The flow rate of the HPLC was set at 0.3 mL/min. The gradient used started with 10% B, increasing linearly to 50% B over 10 min, then to 100% B in 2 min. The mobile phase is held at 100% B for 3 min to purge the system before returning to 10% B for 5 min in preparation for the next injection. The ELSD was set at 65 ± 25°C and 40.0 psi gas pressure. Apramycin sulfate (Sigma Aldrich) was used as standard for peak identification.

The extraction of caerulomycin A from the fermentation of *S. tenebrarius* was based on a method described previously (Zhu et al., 2012). The supernatant was extracted using equal volume ethyl acetate and vigorously shaken. The process was repeated up to three times. The organic fraction was pooled and concentrated using a vacuum concentrator. The residual solid was redissolved in methanol and analyzed by HPLC-MS. The column used was Waters Acquity UPLC BEH C18 Column 1.7 μm (2.1 × 150 mm). The gradient used was the same as used in apramycin quantification, except that the mobile phases were 1% formic acid in water and acetonitrile as A and B, respectively.

The crude extract of BCC24770 was obtained as described previously (She et al., 2020, 2021). Briefly, an equal volume of ethyl acetate was added to the fermentation supernatant and vigorously shaken for 3 h. The mixture was centrifuged at 14,000 *g* for 10 min. The organic phase was pooled in a fresh container. This process was repeated three times in total. Ethyl acetate was evaporated using a vacuum concentrator and the remaining solid was resuspended in methanol and analyzed by HPLC-UV/Vis. The column used was Waters CORTECS™ UPLC C18 1.6 μm (2.1 × 100 mm). The gradient used was the same as used in apramycin quantification, except that the mobile phases were 0.1% trifluoroacetic acid in water and acetonitrile as A and B, respectively.

### Shotgun Proteomics

Shotgun proteomics sample preparation was prepared by a modified acetone precipitation protocol followed by in-solution digestion (Liu et al., 2016; Hao and Lam, 2021). Mycelia were resuspended in lysis buffer (0.5% sodium dodecyl sulfate, 50 mM Tris–HCl, pH 8.5) and incubated at 95°C for 5 min. The suspension was sonicated in an ice bath until translucent and centrifuged at 14,000 *g* for 10 min to remove cell debris. Ice cold acetone was added to the supernatant at a 4:1 ratio and incubated at −20°C overnight. Purified protein was pelleted by centrifugation at 14,000 *g* and 0°C for 30 min and further washed with ice-cold washing solution (80% acetone, 10% methanol, 0.2% acetic acid) to remove sodium dodecyl sulfate attached to the proteins. The protein pellets were then resuspended in 6 M urea, 0.6 M guanidine hydrochloride, and 50 mM ammonium bicarbonate and quantified by bicinchoninic acid assay (Pierce™, Thermo Fisher). A 40 μg aliquots of the protein solution were reduced with 0.1 M dithiothreitol (Sigma) at 37°C for 1 h. Exposed cysteine residues were methylated by 0.2 M iodoacetamide (Sigma) in the dark for 30 min, after which the reaction was quenched using an excess of dithiothreitol. The protein solution was diluted with 50 mM ammonium bicarbonate to a final concentration of 1 M urea. Sequencing-grade modified trypsin (Promega) was used at a 1:50 trypsin to protein ratio. Trypsin digestion was performed overnight at 37°C. Formic acid was added to a final concentration of 0.5% to quench the digestion. The peptide mixture was desalted using C18 ZipTip (Merck Millipore) as per the manufacturer’s instruction. Desalted peptides were stored at −20°C until analysis. Peptides were resuspended in 1% formic acid at 200 ng/μL and injected into Bruker timsTOF Pro Mass-Spectrometer. LCMS operation parameter is listed in Supplementary Table 1.

The LCMS raw data was converted by the msconvert.exe tool of the ProteoWizard suite (Ver. 3.0.21101) (Chambers et al., 2012) to mzXML with the following filters: top 150 most-intense count, 0.05 m/z precursor tolerance, 5 s scan time tolerance, and 0.1 ion mobility tolerance. The *S. coelicolor* A3(2) and BCC24770 protein database used consisted of open reading frames (ORFs) generated by GeneMarkS (Besemer et al., 2001). The *S. tenebrarius* protein database was obtained from the RAST database (Aziz et al., 2008). The genome of *S. coelicolor* A3(2) was obtained from the NCBI Genome database (Bentley et al., 2002). The genome of BCC24770 was sequenced and deposited in a previous study (She et al., 2021). Protein annotation for the *S. coelicolor* A3(2) protein database was performed using BLASTP of the NCBI Blast Suite against *Actinobacteria* proteins downloaded from UniProtKB on 11th November 2021 at 1E-3 *E*-value cutoff (Camacho et al., 2009). Protein identification was performed using the *Trans-*Proteomic Pipeline (Deutsch et al., 2011; Ver, 2021). Peptide-spectrum matches were obtained by sequence database searching using Comet (Ver, 2021). The settings used were precursor peptide mass tolerance of 30 ppm and fragment ion bin tolerance of 0.05. The peptide-spectrum matches were validated by PeptideProphet, iProphet, and ProteinProphet with the false detection rate set at 1%. Label-free quantification was performed by calculating the non-spectral abundance factor (NSAF) (McIlwain et al., 2012) when the average spectral count in a sample group ≥ 3. Proteins that did not pass this threshold or were not detected in the sample were assigned the value of 1/10 of the lowest NSAF.

### Biosynthetic Gene Cluster Identification Algorithm

The fold-change and the *p*-value of pairwise comparisons were calculated using the average NSAF values of each sample group. Differentially expressed proteins were assigned as nodes that scored 10 points when the node was highly differentially expressed (magnitude of the fold-change ≥ 2.0 and *p*-value ≤ 0.01) or five points when the node was moderately differentially expressed (magnitude of the fold-change ≥ 1.5 and *p*-value ≤ 0.05). A positive score was assigned to ORFs that were upregulated at the nutrient-starved phase and a negative score was assigned to ORFs that were expressed during the growth phase. Edges were drawn between nodes that are within 5 ORFs of each other. Each cluster was scored based on the magnitude of the average score of all the nodes. Clusters with scores ≥ 3 are deemed a putative cluster of differentially expressed proteins, hereafter referred to as “proteomining-based clusters” (PBCs) to distinguish from BGCs defined by genome mining. Those with scores between 1.5 and 3 were checked for chimeric clusters by manual inspection. If there exists a partition of the cluster whereby the sub-clusters have score ≥ 3, the chimeric cluster is separated, and the sub-clusters are annotated as a PBC with a letter. These score cutoffs were chosen based on experience with our data. The remaining PBCs containing at least 5 nodes and scored greater than 3 were annotated. Functional analysis of the individual PBCs formed in the proteomics analysis of *S. coelicolor* A3(2) was performed using STRING (Ver. 11.5) with “*Streptomyces coelicolor* A3(2)” as the organism, the “Neighborhood” component of active interaction score disabled, and minimum interaction score set at 0.4 (Medium) (Szklarczyk et al., 2021). The STRING cluster node functions were checked against the STRING database for overall PBC function, if available. BGC characterization was performed by antiSMASH Ver 6.0 using the default settings (Blin et al., 2021). The in-house scripts implementing the proteomining clustering algorithm are available at https://github.com/darwinlinardi/Proteomining-Clustering.

### *Streptomyces tenebrarius* ORF 4229-4230 Knockout

DNA fragments 2,486 bp upstream and 2,436 bp downstream of ORF 4229-4230 (Gene name: *crmA*) was amplified by PCR using the *S. tenebrarius* genome as template. Purified fragments were cloned into the *Hin*dIII*/Eco*RI sites of pWHU258 (Lv et al., 2016) gene knockout vector using Hieff Clone^®^ Plus Multi One Step Cloning Kit (Yeasen, China). The vector was inserted into *S. tenebrarius* via *E. coli*-*Streptomyces* conjugation (Kieser et al., 2000). The resulting in-frame deletion mutants *S. tenebrarius* Δ*crmA* were screened and confirmed by PCR analysis and DNA sequencing. The primers used in the knockout are displayed in Supplementary Table 2.

## Results and Discussion

### *Streptomyces coelicolor* A3(2) Biosynthetic Gene Cluster Proteomining

**FIGURE 1:**
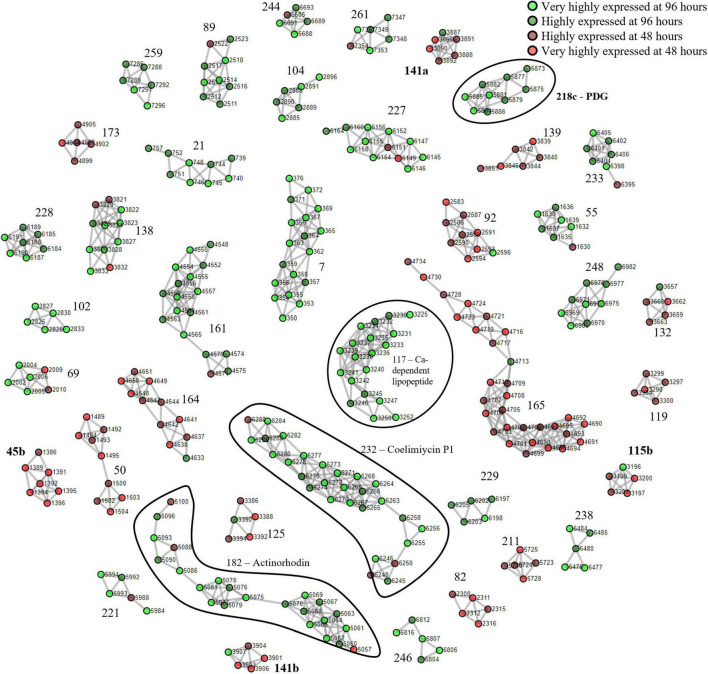
Proteomining result of *S. coelicolor* A3(2) fermentation in GYM medium. 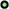 and 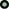 represent proteins that were more abundant in the mycelia harvested at 96 h compared to 48 h at foldchange ≥ 2 and *p*-value ≤ 0.01 (very highly expressed at 96 h), and foldchange ≥ 1.5 and *p*-value ≤ 0.05 (highly expressed at 96 h), respectively. 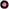 and 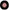 represent proteins that were more abundant in the mycelia harvested at 48 h compared to 96 h at foldchange ≥ 2 and *p*-value ≤ 0.01 (very highly expressed at 48 h), and foldchange ≥ 1.5 and *p*-value ≤ 0.05 (highly expressed at 48 h), respectively. Edges were drawn between nodes with an ORF difference ≤ 5. Cluster numbers in bold indicate clusters that previously scored between 1.5 and 3 which scored ≥ 3 when the chimeric clusters were separated.

We first used *S. coelicolor* A3(2) as a benchmark due to its well-documented expression of BGCs that allowed for clear identification of the clusters. The mycelia harvested at 96 h were designated as the nutrient-starved phase which was indicated by the red pigmentation. The mycelia harvested at 48 h were designated as the growth phase. The described clustering algorithm was applied to yield putative PBCs. The devised PBC scoring system rewarded consistent upregulation of cluster members in the same sample group, divided by the difference between the smallest and largest ORF numbers, to encourage the formation of tight clusters. A total of 57 PBCs had a score ≥ 1.5 and among these, 35 had a score ≥ 3 (Supplementary Figure 1 and Supplementary Data 1). On examination of the cluster structure, 4 of the 22 PBCs with scores between 1.5 and 3 (PBC No. 45, 115, 141, and 218) were decoupled and subsequently formed sub-clusters that scored ≥ 3. In total, 40 PBCs were identified and are displayed in Figure 1. The function of the PBCs was predicted using STRING (Ver 11.5) with the “neighborhood” criterion disabled to increase orthogonality between our proteomining clustering method and STRING since our method had already determined that the proteins in each PBC were already in proximity in the genome (Table 1).

**TABLE 1:** Functional annotation of proteomining clusters of *S. coelicolor* A3(2) at 48 and 96 h.

Cluster	ORF begin	ORF end	Members	Highly expressed at	Cluster function
7	350	376	18	96 h	Polysaccharide biosynthesis
50	1489	1504	9	48 h	Ribosomal proteins
55	1630	1639	7	96 h	Proteasomes
69	2002	2010	6	96 h	Amino acid transport
82	2308	2316	5	48 h	Protein export
89	2511	2523	9	96 h	Sensor
92	2583	2596	9	48 h	Ribosomal proteins
102	2825	2833	5	96 h	Maleate isomerase
115b	3196	3201	5	48 h	Fructose/mannose metabolism
**117**	**3225**	**3252**	**19**	**96 h**	**Ca-dependent lipopeptide biosynthesis**
132	3657	3663	5	48 h	Stress response
141b	3901	3907	5	48 h	Ribosomal proteins
161	4548	4575	17	96 h	NADH-Quinone oxidoreductase
164	4633	4651	11	48 h	Ribosomal proteins
165	4690	4734	30	96 h	Ribosomal proteins
**182**	**5057**	**5100**	**23**	**96 h**	**Actinorhodin biosynthesis**
**218c**	**5873**	**5886**	**9**	**96 h**	**Undecylprodigiosin biosynthesis**
229	6197	6205	5	96 h	Purine metabolism
**232**	**6245**	**6288**	**28**	**96 h**	**Coelimycin P1 biosynthesis**
233	6395	6406	7	96 h	β-alanine metabolism
244	6685	6693	5	96 h	Benzoate degradation
248	6968	6982	9	96 h	Inositol metabolism

*Function annotation of proteomining clusters were performed by STRING (ver. 11.5). BGCs identified by antiSMASH were highlighted in bold. Clusters with unknown function were omitted.*

During the nutrient starvation phase, we detected the upregulation of PBC No. 117, 182, 218c, and 232, annotated by antiSMASH as the calcium-dependent antibiotic, actinorhodin, undecylprodigiosin, and coelimycin P1 BGCs, respectively. The production of calcium-dependent antibiotic, actinorhodin, and undecylprodigiosin under nutrient starvation conditions has been well-documented even before the whole-genome assembly of *S. coelicolor* A3(2) in 2003 (Bentley et al., 2002; Hopwood, 2007; Manteca et al., 2010; Bednarz et al., 2019). However, PBC No. 218c, corresponding to the undecylprodigiosin BGC, required decoupling for the PBC to score ≥ 3. To ensure that this cluster was not falsely identified, we obtained the crude methanolic extract of the mycelia at harvested at 96 h and analyzed it by colorimetry and mass spectrometry (Tenconi et al., 2013). The crude extracts exhibited peak absorption at 230 nm and monoisotopic mass of 394.29, consistent with the undecylprodigiosin standard and confirming the activity of the undecylprodigiosin BGC (Supplementary Figure 2). On the other hand, the production of coelimycin P1 (PBC No. 232) was only recently documented, due to its BGC expression being controlled by a complex regulatory system (Bednarz et al., 2019). As such, we believe that the expression of the coelimycin P1 BGC in our study was triggered by the media composition and fermentation conditions used in this study (Gottelt et al., 2010; Bednarz et al., 2019).

Aside from the PBCs identified as BGCs, several PBCs with various functions, determined by STRING, were also upregulated during nutrient starvation. Prominently, members of PBC No. 161 collectively possessed NADH-quinone oxidoreductase functions. The upregulation of quinone oxidoreductases was reasonable due to its correlation with cellular stress and was hypothesized to be involved in radical build-up relief and detoxification of quinones (Pey et al., 2019). Other PBCs that were upregulated during nutrient starvation possessed polysaccharide biosynthesis (PBC No. 7), amino acid transport (PBC No. 69), sensory (PBC No. 89), maleate isomerase (PBC No. 102), purine metabolism (PBC No. 229), β-alanine metabolism (PBC No. 233), benzoate degradation (PBC No. 244) and inositol metabolism (PBC No. 248) functions. On the other hand, numerous PBCs (PBC No. 50, 92, 141b, 164, and 165) containing ribosomal proteins were downregulated during nutrient starvation, consistent with past proteomic studies of *S. coelicolor* A3(2) (Manteca et al., 2006, 2010; Rioseras et al., 2018). This downregulation is likely attributed to nutrient scavenging attempts by recycling ribosomes (Li et al., 2010). Overall, the proteomining clustering method was capable of highlighting active BGCs as well as other active gene clusters.

### *Streptomyces chrestomyceticus* BCC24770 Biosynthetic Gene Cluster Proteomining

**FIGURE 2:**
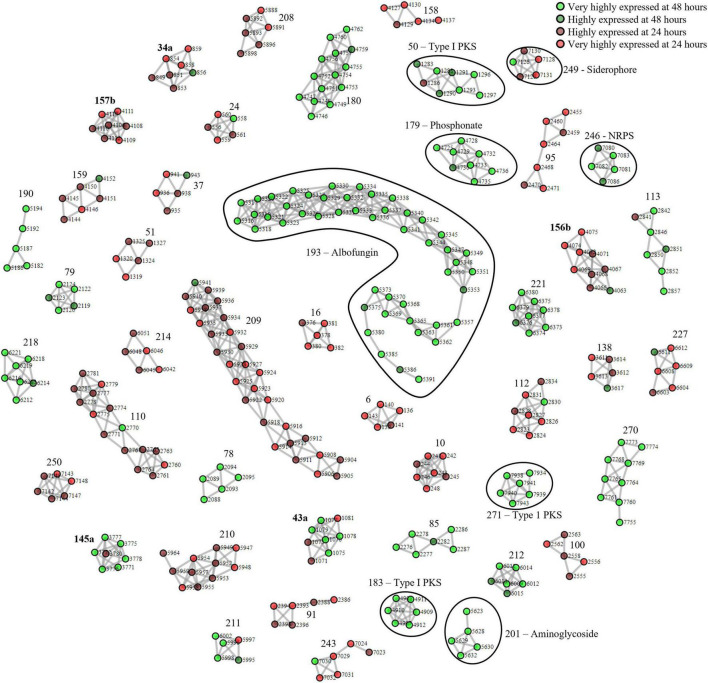
Complete proteomining-based clusters of *S. chrestomyceticus* BCC24770 fermentation in GYM medium with score ≥ 1.5. 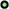 and 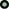
represent proteins that were highly expressed by the mycelia at 48 h (nutrient-starved phase) compared to 24 h (growth phase) at foldchange ≥ 2 and *p*-value ≤ 0.01 (very highly expressed at 48 h), and foldchange ≥ 1.5 and *p*-value ≤ 0.05, respectively, (highly expressed at 48 h). 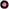 and 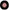 represent proteins that were highly expressed by the mycelia at 24 h (growth phase) compared to 48 h (nutrient-starved phase) at foldchange ≥ 2 and *p*-value ≤ 0.01 (very highly expressed at 24 h), and foldchange ≥ 1.5 and *p*-value ≤ 0.05, respectively, (highly expressed at 24 h). Edges were drawn between nodes with an ORF difference ≤ 5. Cluster numbers in bold indicate clusters that previously scored between 1.5 and 3 which scored ≥ 3 when the chimeric clusters were separated.

Next, we applied our method to *S. chrestomyceticus* BCC24770, which produced a variety of natural products with bioactivity against various nosocomial pathogens (She et al., 2020, 2021). Since the genome of BCC24770 was not fully assembled, being comprised of 70 contigs, we aimed to use this organism to test our proteomining method. Also, unlike *S. coelicolor* A3(2) which exhibited red pigmentation due to accumulation of undecylprodigiosin, the fermentation of BCC24770 did not exhibit detectable cues that indicated production of secondary metabolites. As such the production of albofungin (Supplementary Figure 3) was used as the indicator for the nutrient-starved and growth phase, respectively, which were the mycelia harvested from the fermentation in GYM at 48 and 24 h, respectively. The proteomining clustering result yielded 45 PBCs post-decoupling (Figure 2 and Supplementary Data 2). The annotation of detected PBCs was performed by consolidating the function of each ORF in the PBC (Table 2). Like the proteomics analysis of *S. coelicolor* A3(2), there was an upregulation in NADH-quinone oxidoreductase activity (PBC No. 212) and a significant downregulation in ribosomal proteins and housekeeping proteins (PBC No. 10, 91, 95, 208–210, and 250).

**TABLE 2:** Functional annotation of proteomining clusters of fermentation mycelia *S. chrestomyceticus* BCC24770 in GYM broth at 24 and 48 h.

Cluster	Highly expressed at	Cluster function	Similar BGCs (Top 3)
6	24 h	Iron-sulfur binding proteins	
10	24 h	Cell division-related proteins	
**50**	**48 h**	**Type I PKS**	**Cycloheximide (27%)** **9-Methylstreptimidone (19%)**
91	24 h	Secretion proteins	
95	24 h	Housekeeping proteins	
110	24 h	ATP Synthases	
**179**	**48 h**	**Phosphonate**	**Dehydrophos (11%)**
**183**	**48 h**	**NRPS**	**Piericidin A1 (50%)**
**193**	**48 h**	**Type II PKS (Albofungin)**	
**201**	**48 h**	Aminoglycoside*	**Neomycin (85%)** **Paromomycin (82%)**
208	24 h	Ribosomal proteins/tRNA dimerization	
209	24 h	Ribosomal proteins	
210	24 h	Ribosomal proteins	
212	48 h	NADH-quinone oxidoreductase	
**246**	**48 h**	**NRPS**	**Mannopeptimycin (81%)**
**249**	**24 h**	**NRPS (Siderophore)**	**Paenibactin (83%)** **Streptobactin (70%)** **Bacillibactin (38%)**
250	24 h	Isochorismate metabolism	
270	48 h	Glycosyl/methyltransferases	
**271**	**48 h**	**Type I PKS**	**Piericidin A1 (58%)**

BGCs were identified by antiSMASH and is highlighted in bold. * – Only 1 core biosynthetic cluster was differentially expressed between the two sample groups.

*Clusters with unknown functions were omitted.*

The PBCs identified were then compared to the genome mining result of antiSMASH. We independently identified PBC No. 193, the largest PBC, as a type II PKS similar to xantholipin BGC. This type II PKS BGC has been characterized to produce albofungin by She et al. Most of the genes in this BGC were detected only during nutrient starvation, except Alb66 (ORF No. 5370. Function: FAD-binding monooxygenase) which was present in both sample groups but still highly upregulated during nutrient starvation (Supplementary Table 3). Several transcriptional regulator proteins (ORF No. 5327, 5350, and 5361 corresponding to Alb22, Alb45, and Alb57, respectively) were also only detected during nutrient starvation, suggesting their involvement in eliciting albofungin production.

Aside from the albofungin BGC, the expression of several smaller orphan BGCs were also detected (Table 2). Two type I PKS (PBC No. 50 and 271), 2 NRPS (PBC No. 183 and 246), and 1 phosphonate BGCs (PBC No. 179) were upregulated at the nutrient-starved phase, while PBC No. 249, a putative NRPS BGC that produced siderophores, was the only downregulated BGC. The smaller size of these PBCs, compared to PBC No. 193 or the PBCs identified from *S. coelicolor* A3(2), could be due to the smaller size of the BGCs or inherently lower production of the respective compounds at the time of sampling. Notably, PBC No. 183 and 271 were located near the edge of their respective contigs, as such the smaller size of these 2 PBCs was likely due to an incomplete BGC sequence. Future work could focus on integrating metabolomics in the detection of the products of these orphan BGCs.

To obtain differing fermentation conditions with potentially different secondary metabolite production profiles, we performed fermentation of BCC24770 in TSBY. The fermentation in TSBY was selected due to a stark difference in albofungin production (192–282-fold decrease), which could lead to a different main product of the fermentation (Supplementary Figure 3). We then took this opportunity to compare the PBCs identified from the proteomining of BCC24770 fermented in different media to the PBCs identified from the nutrient-starved/growth comparison. Surprisingly, the loss of albofungin production in the fermentation of BCC24770 in TSBY did not result in increased production of other secondary metabolites (Supplementary Figure 4 and Supplementary Data 3). In fact, the PBCs obtained when comparing BCC24770 fermentation in different media were very similar to the nutrient-starved/growth. This suggests a significant downregulation of secondary metabolite biosynthesis of the fermentation in TSBY since some proteins of the albofungin BGC were still expressed at very low concentrations. Some PBCs were also not detected in the proteomining of BCC24770 fermentation in different media, namely a siderophore producing PBC and a putative phosphonate BGC as well as some ribosomal protein PBCs. This could be attributed to a similar trend of downregulation in the mycelia as the culture reaches the stationary phase such as the recycling of ribosomal proteins (Li et al., 2010).

### *Streptomyces tenebrarius* Biosynthetic Gene Cluster Proteomining

**FIGURE 3:**
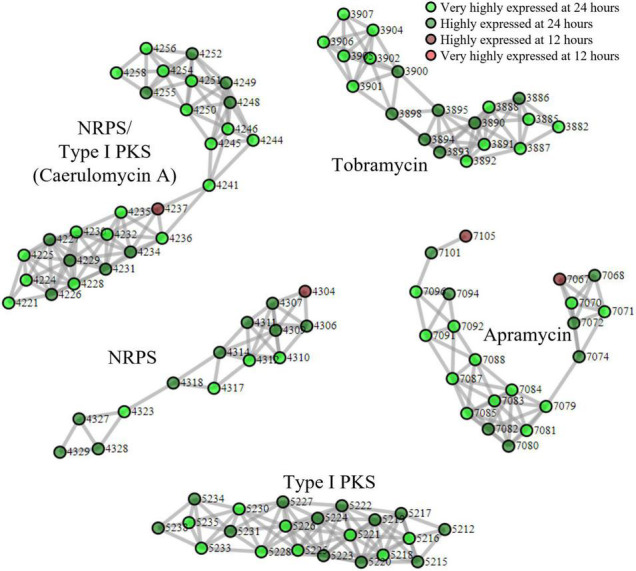
Proteomining result of *S. tenebrarius* fermentation in TSBY medium. 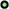 and 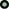 represent proteins that were more abundant in the mycelia harvested at 24 h compared to 12 h at foldchange ≥ 2 and *p*-value ≤ 0.01 (very highly expressed at 24 h), and foldchange ≥ 1.5 and *p*-value ≤ 0.05 (highly expressed at 24 h), respectively. 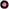 and 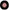 represent proteins that were more abundant in the mycelia harvested at 12 h compared to 24 h at foldchange ≥ 2 and *p*-value ≤ 0.01 (very highly expressed at 12 h), and foldchange ≥ 1.5 and *p*-value ≤ 0.05 (highly expressed at 12 h), respectively. Edges were drawn between nodes with an ORF difference ≤ 5. Cluster score ≥ 3.

Finally, we applied our proteomining clustering algorithm to the apramycin producer, *S. tenebrarius*. Similar to the fermentation of BCC24770, the growth state of *S. tenebrarius* was monitored by the quantity of apramycin in the supernatant (Supplementary Figure 5). Apramycin production started between the 12th and 18th hour of fermentation with peak apramycin production between the 18th and 24th hour and plateauing by the 48th hour. The time points 24 and 12 h were appointed as the peak secondary metabolite production phase and the non-producing growth phase, respectively. Figure 3 displays all the PBCs detected with scores ≥ 3 (Supplementary Data 4). Interestingly, only 5 PBCs were expressed, all of which were identified by antiSMASH as BGCs.

**FIGURE 4:**
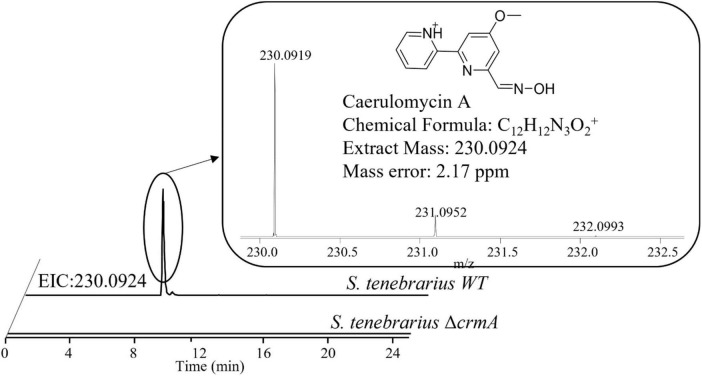
HPLC-MS identification of caerulomycin A from the crude extract of fermentation supernatant of *S. tenebrarius* WT and Δ*crmA*. The extracted ion chromatogram (EIC) was obtained ions with m/z of 230.0924, corresponding to singly protonated caerulomycin A.

Aside from the expression of apramycin BGC, which was monitored throughout the fermentation, the expression of the tobramycin BGC was not unexpected as it has been documented in previous studies of *S. tenebrarius* (Borodina et al., 2005; Hong and Yan, 2012). Interestingly, we also detected the expression of 1 NRPS/Type I PKS hybrid, 1 NRPS and 1 type I PKS BGC which had not been documented in this species before. The closest match for these BGCs were the caerulomycin A (56% similarity), mannopeptimycin (33%) and spectinabilin (81%) BGCs, respectively. We then sought to isolate one of the products of the active BGCs utilizing the ethyl acetate extraction method employed by Zhu et al. (2012) to isolate caerulomycin A. HPLC-MS analysis detected a compound with a mass of 230.09 by HPLC-MS, corresponding to caerulomycin A (Figure 4). Here, we demonstrated the use of proteomics to guide secondary metabolite isolation.

Since there was no previous report of the production of caerulomycin A in *S. tenebrarius*, we sought to further confirm the role of the discovered caerulomycin A BGC by gene knockout. We proceeded to knock out ORF 4229–4230 which corresponded to *crmA*, the core biosynthetic protein for caerulomycin A biosynthesis, to probe how this BGC affects secondary metabolite production of *S. tenebrarius*. The loss of *crmA* abolished caerulomycin A production in *S. tenebrarius* (Figure 4). Surprisingly, knocking out *crmA* also abolished apramycin production (Supplementary Figure 5). We then took this opportunity to test our proteomining clustering method on strain comparison, by comparing *S. tenebrarius* Δ*crmA* and the WT (Supplementary Figure 6 and Supplementary Data 5). Differential expression analysis yielded the same 5 PBCs as the nutrient-starved/growth phase comparison, indicating overall downregulation of secondary metabolite biosynthesis due to the loss of *crmA*. The mechanism behind this cross-BGC regulation is still unclear. Since *crmA* is not a regulatory gene but rather a NRPS that contained a ketosynthase, acyltransferase, acyl-carrier, condensation, adenylation and thiolation domains, this made it unlikely that the overall downregulation of the other BGCs was due to cross-BGC regulation. As such, we hypothesize that the loss of the secondary metabolite BGC expression may be attributed to the loss of caerulomycin A or one of its precursors, which might act as the trigger for secondary metabolite production either by triggering programmed cell death (Tenconi et al., 2018) or through quorum sensing (Koberska et al., 2021).

## Conclusion

In summary, we developed a proteomining method based on label-free quantitative proteomics of *Streptomyces* sp. We first showed that the different phases of the *Streptomyces* spp. life cycle can be exploited to generate contrasting producing/non-producing states, as demonstrated in a proof of concept in *S. coelicolor* A3(2). Furthermore, the increase in production of secondary metabolite under nutrient starvation conditions is a widespread phenomenon (Hopwood, 2007; Rigali et al., 2008). This approach reduces the need for formulating different fermentation conditions or prior genetic manipulation to produce such contrast, although these other comparisons can also be used.

In addition, recent advancements in LCMS-based mass spectrometry sample preparation techniques and instruments has allowed label-free quantitative proteomics to sufficiently highlight the numerous differentially expressed proteins, while also having the advantages of lower costs, simpler experiments, and the ability to compare across more sample groups, unlike isobaric labeling methods (Du and van Wezel, 2018). Our simple algorithm for clustering differentially expressed proteins by proximity in the genome also facilitated the automated and independent identification of potential BGCs. Compared to the method developed by Gubbens et al. (2014) our algorithm integrates gene proximity and protein differential expression to highlight BGCs more systematically with our scoring system and directly from label-free quantitative proteomics data, packaged into a simple script. Furthermore, our workflow is simpler, requiring only a single pair of comparison. Overall, our proposed clustering-based proteomining method should be able to detect BGCs with less bias due to non-reliance on co-factors or high molecular weights of proteins (Meluzzi et al., 2008; Bumpus et al., 2009; Chen et al., 2012). Therefore, our proposed workflow, when combined with traditional genome mining, should be helpful in guiding the researcher to isolate novel secondary metabolites produced by *Streptomyces* spp.

In terms of limitations, like other expression-based analysis, proteomining is only applicable when the *Streptomyces* strain is culturable under laboratory conditions and typically relies on nutrient starvation to trigger the production of secondary metabolites (Rigali et al., 2008). In the future, our proteomining clustering algorithm could also be applied in conjunction of the use of engineered media composition or specialized fermentation setup to better simulate the natural habitat of *Streptomyces* sp. which can elicit the expression of silent BGCs (Du and van Wezel, 2018). Another complication is the tendency for *Streptomyces sp.* to activate multiple BGCs to produce distinct secondary metabolites under similar conditions. While the pool of BGC candidates would have been narrower compared to the total BGCs in the genome, it can still be difficult to unambiguously link the BGC to a secondary metabolite of interest based on proteomining alone, though tools such as antiSMASH, PRISM and BLASTP may be called upon to help narrow down the possibilities. In the future, it will be worthwhile to combine time-series metabolomics with proteomics to reveal temporal correlations between individual secondary metabolite production and protein expression changes, to expedite the association of the BGCs to their products with higher confidence and throughput.

## Data Availability Statement

The datasets presented in this study can be found in online repositories. The names of the repository/repositories and accession number(s) can be found below: http://www.proteomexchange.org/, PXD032774; http://www.proteomexchange.org/, PXD032775; http://www.proteomexchange.org/, PXD033654. All datasets have been made public.

## Author Contributions

DL and WS designed and performed the experiments and analyzed the data. QZ performed the gene knockout and caerulomycin A isolation experiments in *S. tenebrarius*. DL wrote the manuscript. HL, YY, and P-YQ supervised the project. All authors revised and edited the manuscript.

## Conflict of Interest

The authors declare that the research was conducted in the absence of any commercial or financial relationships that could be construed as a potential conflict of interest.

## Publisher’s Note

All claims expressed in this article are solely those of the authors and do not necessarily represent those of their affiliated organizations, or those of the publisher, the editors and the reviewers. Any product that may be evaluated in this article, or claim that may be made by its manufacturer, is not guaranteed or endorsed by the publisher.
