# Supplementary material for: Proteomining-Based Elucidation of Natural Product Biosynthetic Pathways in Streptomyces
Source: Front Microbiol. 2022 Jul 11;13:913756. doi: 10.3389/fmicb.2022.913756 (PMC9309509; doi:10.3389/fmicb.2022.913756)
Supplement: Supplementary file 1 [file Data_Sheet_1.PDF]

## Table of contents:

**Supplementary Table S1**. LCMS operation parameter. 2

**Supplementary Table S2**. Primers for ORF 4229-4230 (*crmA*) knockout. 2

**Supplementary Table S3**. Albofungin BGC protein list. 3

**Supplementary Figure S1**. Proteomining clusters of *S. coelicolor* with score ≥ 1.5. 4

**Supplementary Figure S2**. Undecylprodigiosin characterization. 5

**Supplementary Figure S3**. Albofungin quantification. 5

**Supplementary Figure S4**. Proteomining clusters of BCC24770 media comparison. 6

**Supplementary Figure S5**. Time course apramycin production. 7

**Supplementary Figure S6**. Proteomining clusters of *S. tenebrarius* mutant comparison. 8

**Supplementary Table S1**. Shotgun proteomics LCMS analysis operation instruments and parameters.

| | | |
|---|---|---|
| LC Module | Bruker nanoElute | |
| LC Column | Ion Optiks Aurora series emitter C18 column with CSI (25cm x 75µm ID, 1.6 µm) | |
| Mobile Phase A | 98% Water, 2% acetonitrile, 0.1% formic acid | |
| Mobile Phase B | 100% acetonitrile, 0.1% formic acid | |
| Column Temperature | | 50°C |
| Flow Rate | | 0.3µL/min |
| | Time | Composition (%B) |
| Gradient | 0.00 | 2.0 |
| | 0.50 | 5.0 |
| | 27.00 | 30.0 |
| | 27.50 | 95.0 |
| | 28.00 | 95.0 |
| | 28.10 | 2.0 |
| | 30.00 | 2.0 |
| Mass spectra peak detection | Use maximum intensity<br>Absolute threshold = 10 | |
| Ion polarity | Positive | |
| Scan range (m/z) | | 100-1700 |
| 1/k0 ($V \cdot s/cm^2$) | | 0.85-1.30 |
| Ion Source | Capillary (V) | 1400 |
| | Dry gas (l/min) | 3.0 |
| | Dry temp (°C) | 180 |

**Supplementary Table S2**. Primers used for ORF 4229-4230 knock out

| Primer | Sequence | Function |
|---|---|---|
| *orf4229-4230-*left-F | GTAAAACGACGGCCAGTGCCAAGCTTGTGATCGCGACACCGCAGACGTACAAGGCG | *orf4229-4230* in-frame deletion |
| *orf4229-4230-*left-R | GTTGATGTTGAAGAACGCGGCGTCGAACAG | *orf4229-4230* in-frame deletion |
| *orf4229-4230-right-F* | CTGTTCGACGCCGCGTTCTTCAACATCAACCGCCGGGCCGAGCGGGGTCACCGCGCC | *orf4229-4230* in-frame deletion |
| *orf4229-4230-right-R* | AACAGCTATGACATGATTACGAATTCGTCGGGGTCTGGCTGCACATCGTGACCTTC | *orf4229-4230* in-frame deletion |

**Supplementary Table S3**. Protein expression of predicted gene of the albofungin BGC (Cluster 193).

| Protein in previous study | ORF No | Protein function | Fold-change | p-value |
|---|---|---|---|---|
| Alb36 | 5341 | PKS | Only at 48H | |
| Alb37 | 5342 | PKS | Only at 48H | |
| Alb44 | 5349 | PKS | Only at 48H | |
| Alb26 | 5331 | Oxidoreductase | Only at 48H | |
| Alb33 | 5338 | Oxidoreductase | Only at 48H | |
| Alb43 | 5348 | Oxidoreductase | Only at 48H | |
| Alb35 | 5340 | Cyclase | Only at 48H | |
| Alb48 | 5353 | Cyclase | Only at 48H | |
| Alb39 | 5344 | Cyclase | Only at 48H | |
| Alb25 | 5330 | Monooxygenase | Only at 48H | |
| Alb59 | 5363 | O-methyltransferase | Only at 48H | |
| Alb60 | 5364 | O-methyltransferase | Only at 48H | |
| Alb23 | 5328 | FAD-binding monooxygenase | Only at 48H | |
| Alb27 | 5332 | FAD-binding monooxygenase | Only at 48H | |
| Alb66 | 5370 | FAD-binding monooxygenase | 6.0 | 9.4E-04 |
| Alb21 | 5326 | Asparagine synthase | Only at 48H | |
| Alb61 | 5365 | Cytochrome P450 | Only at 48H | |
| Alb19 | 5324 | Halogenase | Only at 48H | |

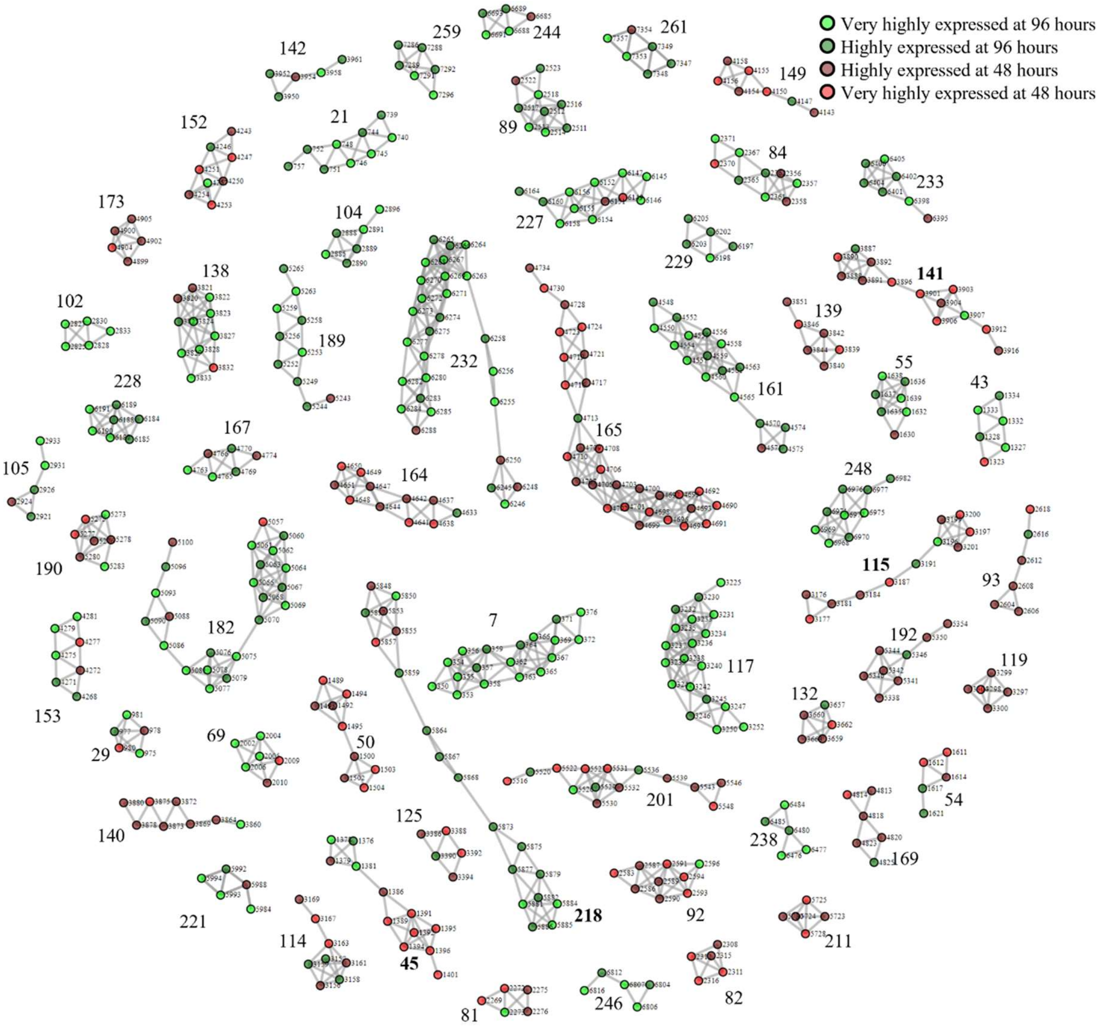


**Supplementary Figure S1**. Complete proteomining-based clusters of *S. coelicolor* A3(2) fermentation in GYM medium with score ≥ 1.5. and represent proteins that were highly expressed by the mycelia at 96 hours (nutrient-starved phase) compared to 48 hours (growth phase) at foldchange ≥ 2 and p-value ≤ 0.01, and foldchange ≥ 1.5 and p-value ≤ 0.05 respectively. and represent proteins that were highly expressed by the mycelia at 48 hours (growth phase) compared to 96 hours (nutrient-starved phase) at foldchange ≥ 2 and p-value ≤ 0.01, and foldchange ≥ 1.5 and p-value ≤ 0.05 respectively.

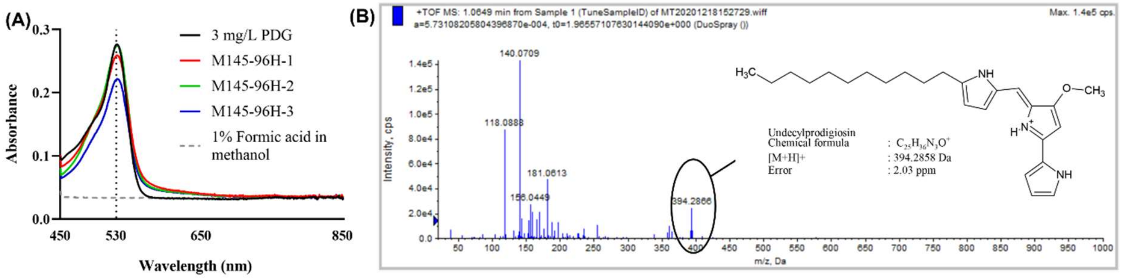


**Supplementary Figure S2**. Characterization of undecylprodigiosin. (A) Light absorbance spectrum of pure undecylprodigiosin and the acidic methanol extract of mycelia. (B) MS spectrum of the acidic methanol extracts.

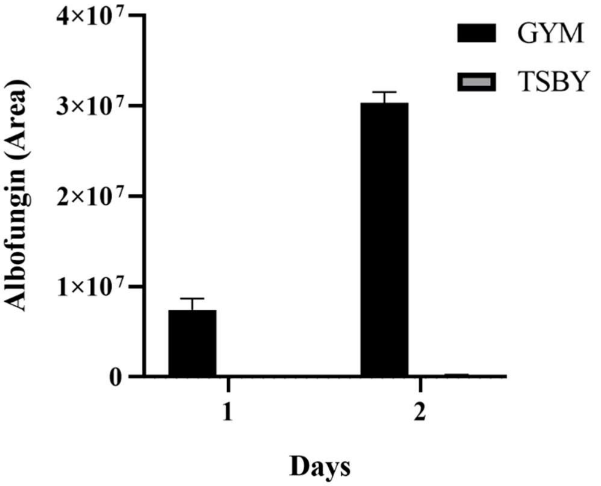


**Supplementary Figure S3**. Albofungin production of BCC24770 in GYM and TSBY fermentation.

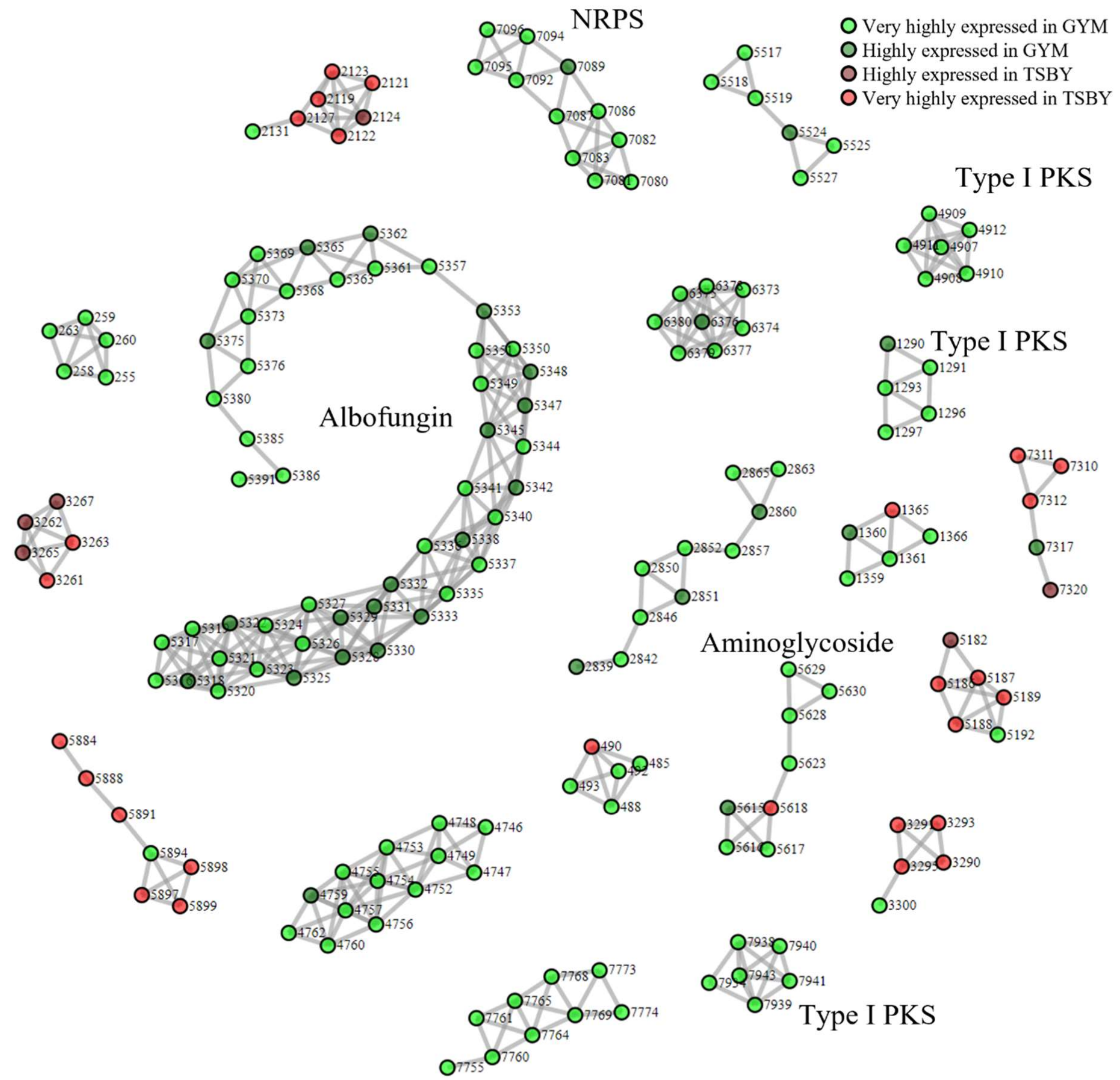


**Supplementary Figure S4**. Proteomining-based clusters of *S. chrestomyceticus* BCC24770 fermentation in GYM medium compared to TSBY medium at 48 hours with score ≥ 3. and represent proteins that were highly expressed by the mycelia in GYM compared to TSBY at foldchange ≥ 2 and p-value ≤ 0.01, and foldchange ≥ 1.5 and p-value ≤ 0.05 respectively. and represent proteins that were highly expressed by the mycelia in GYM compared to TSBY at foldchange ≥ 2 and p-value ≤ 0.01, and foldchange ≥ 1.5 and p-value ≤ 0.05 respectively.

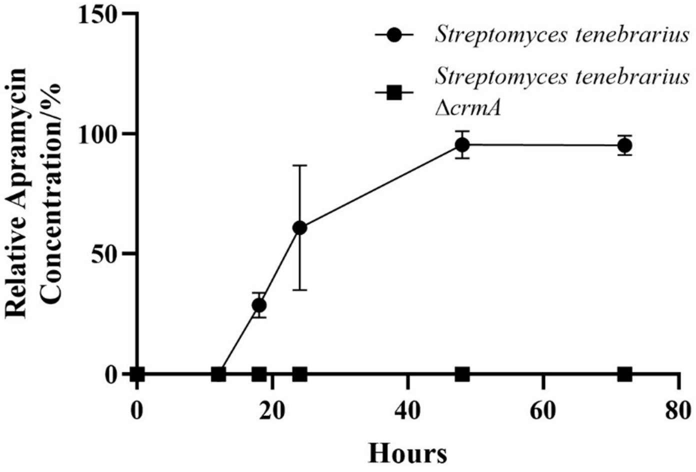


**Supplementary Figure S5**. HPLC-ELSD quantification of apramycin concentration in the fermentation supernatant of *S. tenebrarius* WT *and* Δ*crmA*. Apramycin sulfate was used as standard.

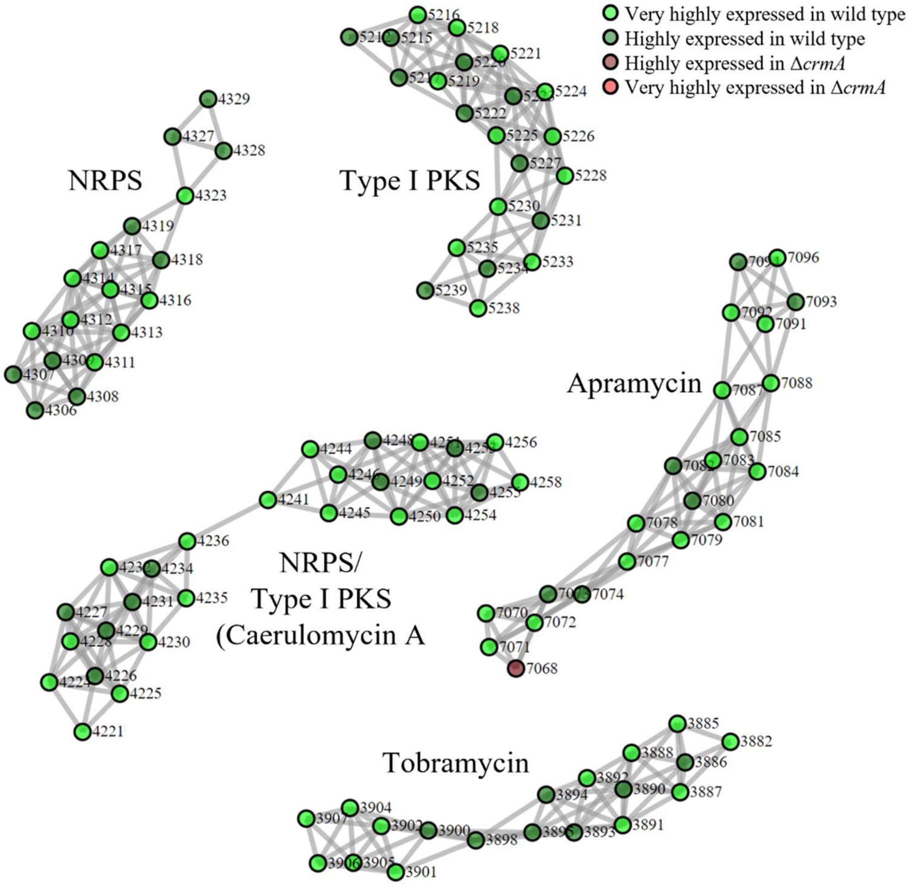


**Supplementary Figure S6**. Proteomining-based clusters of TSBY fermentation of *S. tenebrarius* WT compared to *S. tenebrarius* Δ*crmA* with score ≥ 1.5. and represent proteins that were highly expressed in the mycelia of *S. tenebrarius* WT compared to *S. tenebrarius* Δ*crmA* at foldchange ≥ 2 and p-value ≤ 0.01, and foldchange ≥ 1.5 and p-value ≤ 0.05 respectively. and represent proteins that were highly expressed in the mycelia of *S. tenebrarius* Δ4229-4232 compared to *S. tenebrarius* WT at foldchange ≥ 2 and p-value ≤ 0.01, and foldchange ≥ 1.5 and p-value ≤ 0.05 respectively. Only clusters with scores ≥ 3 were displayed.
